# Effects of hydrotherapy and hammock positioning on pain reduction in preterm neonates

**DOI:** 10.1016/j.jped.2026.101503

**Published:** 2026-02-06

**Authors:** Ana Talita Vasconcelos Arcanjo Ribeiro da Silva, Francisco Plácido Nogueira Arcanjo, Jeferson de Sousa Justino, Lizandro de Andrade Teles, Ana Kelly Melo de Aquino

**Affiliations:** Universidade Federal do Ceará, Programa de Pós-Graduação em Ciências da Saúde, Sobral, CE, Brazil

**Keywords:** Preterm neonates, Neonatal pain, Hydrotherapy, Hammock positioning, NFCS, Non-pharmacological analgesia

## Abstract

**Objective:**

To evaluate the effects of hydrotherapy and hammock positioning, applied individually or in combination, on pain reduction in preterm neonates.

**Methods:**

This randomized clinical trial included 45 preterm neonates under 37 weeks of gestation and weighing less than 2,500g, admitted to the neonatal intensive care unit or intermediate care unit at Santa Casa Hospital in Sobral, Brazil. Palliative interventions were performed during routine invasive and painful procedures, specifically heel lances and handling of the orogastric tube during feeding. Neonates were randomized into three groups: hydrotherapy, hammock positioning, or a combined intervention, with 15 participants per group. Interventions were applied once daily for 15 consecutive days. Pain was assessed using the Neonatal Facial Coding System before and after each session, totaling 1,350 evaluations. Continuous variables were analyzed using Student’s t-test, and categorical variables were analyzed using Fisher’s exact test. The study was registered in the Brazilian Registry of Clinical Trials.

**Results:**

The sample consisted of 53.3% male neonates, with a mean gestational age of 32.4±2.1 weeks. Hydrotherapy significantly reduced pain scores and pain prevalence. Hammock positioning also produced significant reductions in pain scores and prevalence. The combined intervention resulted in the greatest reduction in pain scores and pain prevalence. All within-group comparisons showed statistically significant improvements. No statistically significant differences were observed when the three intervention groups were compared directly.

**Conclusion:**

Hydrotherapy and hammock positioning, applied individually or in combination, are effective, safe, and feasible strategies for reducing pain in preterm neonates and may complement standard neonatal care in NICUs.

## Introduction

Preterm birth, defined by the World Health Organization as delivery before 37 completed weeks of gestation [1], confers marked vulnerability due to physiological immaturity and enduring misconceptions, such as the belief that neonates, particularly preterm infants, do not fully experience pain. These notions have been unequivocally refuted, with evidence showing that even very premature infants perceive and respond to painful stimuli [2].

The perception of pain in preterm infants is complex and clinically significant. The central nervous system begins to develop early in gestation, and although the nociceptive system is not fully mature at birth, it is functional from as early as 20–24 weeks of gestation [3,4]. Peripheral nociceptors and ascending pathways are active at this stage, but inhibitory circuits remain immature, resulting in heightened sensitivity to painful stimuli. The gradual process of myelination and the ongoing maturation of brain structures such as the thalamus and somatosensory cortex further influence how pain is processed. Consequently, preterm infants may display exaggerated or prolonged responses to pain when compared to full-term infants [3].

Pain in the neonatal period is not only perceived by newborns, including those born preterm, but also elicits significant physiological and neuroendocrine responses with both immediate and long-lasting consequences. These responses include alterations in heart rate, oxygen saturation, blood pressure, hormonal profiles, and metabolic activity [5]. Acutely, repeated painful experiences may compromise neuronal development, modulate stress reactivity, and influence subsequent behavioral responses, reinforcing the definition of pain as an unpleasant sensory and emotional experience linked to actual or potential tissue injury [5,6]. Recent evidence has demonstrated that early neonatal pain exposure is associated with altered physiological outcomes, including later dysregulation of cortisol levels in childhood. In neonatal intensive care units (NICUs), preterm infants are frequently subjected to multiple invasive procedures that may generate pain, leading to behavioral stress and adverse physiological outcomes [[7], [8], [9]].

Accurate and systematic pain assessment is therefore essential for ensuring safe and effective pain management in preterm infants. More than 40 neonatal pain assessment tools have been developed, yet only a few are routinely applied in clinical practice. Among these, the Neonatal Facial Coding System (NFCS) is one of the most well-established and widely used instruments. It evaluates facial expressions as indicators of pain, and scores of 3 or higher (NFCS ≥ 3) are typically interpreted as evidence of pain [10,11].

While pharmacological treatments remain central to neonatal pain management, non-pharmacological strategies have been increasingly recognized as valuable adjuncts. Hydrotherapy is a therapeutic intervention involving controlled immersion in warm water, which promotes muscle relaxation, reduces stress responses, and supports autonomic regulation by simulating the intrauterine environment. Hammock positioning (redotherapy) is a positioning technique designed to provide postural containment and flexion, facilitating physiological stability and behavioral organization. Both interventions aim to support neonatal homeostasis, defined as the ability to maintain stable cardiorespiratory, thermal, and neurobehavioral functions in response to environmental stressors. These techniques have been increasingly employed in preterm infants to reduce stress, improve comfort, and provide analgesic effects [[12], [13], [14], [15]].

Despite growing interest in these non-pharmacological approaches, there remains a lack of randomized clinical trials directly comparing the isolated and combined effects of hydrotherapy and hammock positioning on pain reduction during routine painful procedures in preterm neonates. This gap underscores the necessity of conducting the present study.

Given this context, the present study seeks to evaluate the effects of hydrotherapy and hammock positioning, both individually and in combination, on the reduction of pain in hospitalized preterm neonates.

## Methods

### Study design and ethical approval

This study was a randomized clinical trial conducted at Santa Casa de Misericórdia Hospital in Sobral, Ceará, Brazil. Data collection and intervention procedures occurred between July 2022 and October 2023. The trial was approved by the Research Ethics Committee on Human Beings (CAAE 89955118.3.0000.8109) and registered in the Brazilian Clinical Trials Registry (ReBEC; RBR-6g5f4jz).

### Participants

Eligible participants were preterm neonates admitted to the Neonatal Intensive Care Unit (NICU) or the Intermediate Care Unit (IMCU). Inclusion criteria were gestational age below 37 weeks, birth weight under 2,500 g, clinical stability, and more than five days of life. Neonates were required to be receiving exclusive or partial breastfeeding, hydrolyzed milk, or a combination of these**,** with fractional enteral feeding administered every three hours via an orogastric tube.

Neonates were excluded if they were discharged before completing the 15-day intervention, presented with clinical instability during the study, demonstrated feeding intolerance or gastrointestinal disturbances such as diarrhea or vomiting for three consecutive days, or required invasive or non-invasive mechanical ventilation or supplemental oxygen. Additional exclusion criteria included the use of phototherapy, congenital infections, newly diagnosed neurological or genetic syndromes, fasting, central venous access, or a history of surgery during the intervention period.

### Sample size and recruitment

Sample size was calculated a priori based on expected differences in Neonatal Facial Coding System (NFCS) scores before and after non-pharmacological interventions, using data from previous studies evaluating behavioral pain outcomes in preterm neonates. Assuming a moderate effect size (Cohen’s d = 0.6), a two-sided alpha level of 0.05, and a statistical power of 80%, the minimum required sample size was estimated at 13 neonates per group (G*Power software, version 3.1). To account for potential attrition and clinical instability, the sample size was increased to 15 neonates per group, resulting in a total sample of 45 participants, which is consistent with sample sizes used in similar neonatal interventional trials.

Over a 16-month recruitment period, 57 preterm neonates with low birth weight met the inclusion criteria. Twelve were excluded for meeting at least one exclusion criterion, leaving 45 participants. These neonates were randomized into three groups of 15 each.

### Randomization and allocation

Randomization was performed using an online software tool (www.randomizer.com). Because the number of eligible neonates available concurrently was limited, allocation and data collection were conducted gradually and prospectively.

#### Timing and clinical context of interventions

All palliative interventions were performed during periods of routine neonatal care and were not synchronized with acute invasive procedures such as heel lance, venipuncture, or catheter insertion. The objective was to evaluate baseline and ongoing pain-related behavioral stress responses associated with hospitalization, handling, positioning, and routine caregiving, rather than procedural pain. Interventions were consistently administered at the same time each day, approximately 30–60 minutes after feeding and clinical stabilization, to minimize confounding effects related to hunger, medical manipulation, or acute discomfort.

In the present study, the term *palliative interventions* refers to non-pharmacological, non-invasive, and supportive care strategies aimed at alleviating pain, stress, and discomfort in preterm neonates during hospitalization. These interventions are intended to promote comfort, physiological stability, and behavioral organization, without curative intent and without association with end-of-life care. Within this context, hydrotherapy and hammock positioning were considered palliative measures because they focus on symptom relief and improvement of neonatal well-being during routine care.

### Interventions

Participants were assigned to one of three groups: hydrotherapy only, hammock positioning only, or a combined intervention using both techniques. Each intervention was applied once daily for 15 consecutive days.

**Figure 1:**
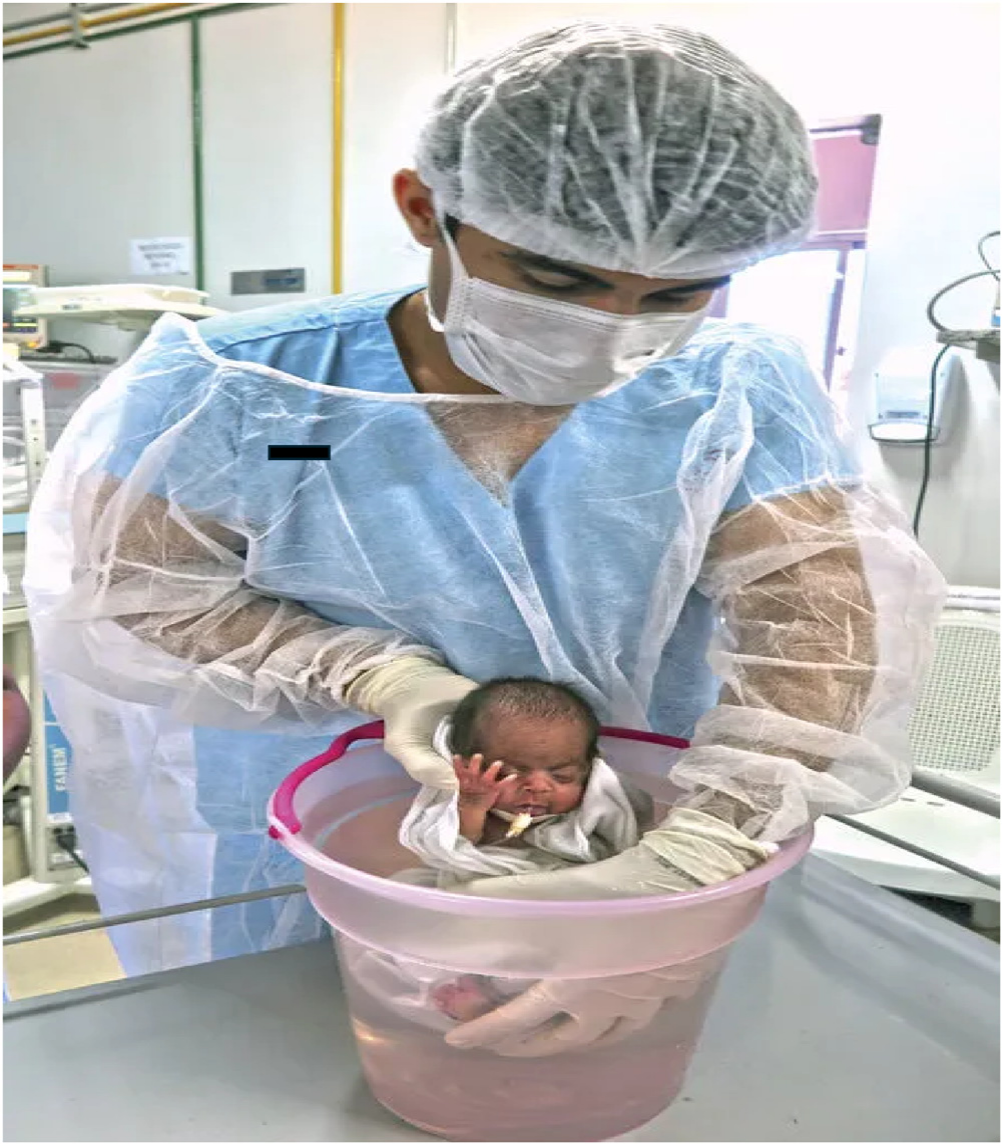
Application of hydrotherapy (source: authors’ collection).

The hydrotherapy protocol consisted of wrapping each neonate in a 1 m^2^ cotton cloth to simulate intrauterine flexion, followed by immersion up to the suprasternal notch in 37°C water in a 15-liter plastic basin for 15 minutes. Neonates were stabilized with hands placed at the cervical and sacral regions, allowing a seated position and free movement while keeping the head above water. After 15 minutes, they were dried with cotton towels and returned to incubators or cribs. Basins were sanitized daily with neutral soap (Figure 1).

**Figure 2:**
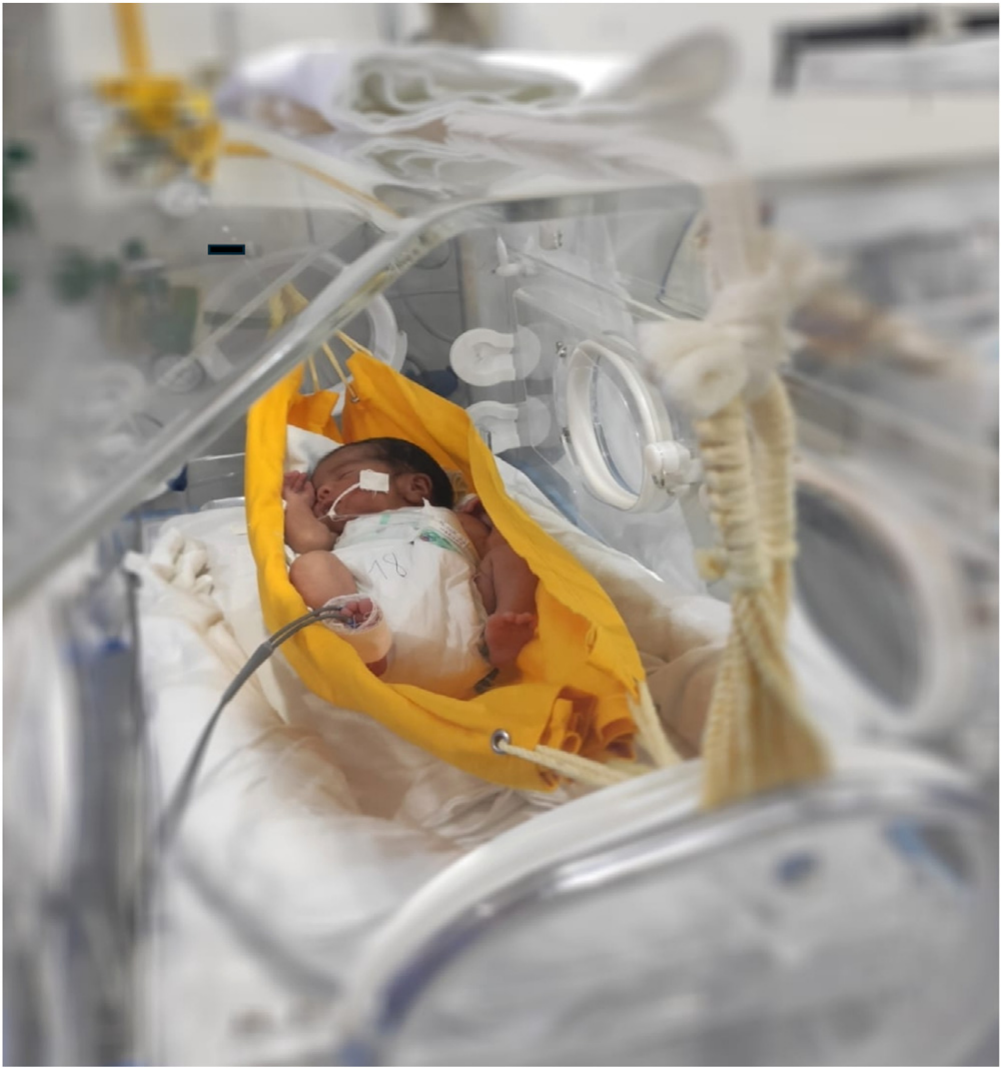
Application of hammock positioning (source: authors’ collection).

The hammock positioning protocol was performed inside incubators, suspending a 100% cotton hammock (3 mm thick, 50 cm wide, 65 cm long) at 10 cm above the mattress. Neonates were placed in the supine position for 120 minutes, with additional cloth support under the scapular and cervical regions to avoid excessive neck flexion. Safety measures included leaving orogastric tubes open when present and continuous monitoring of oxygen saturation and pulse using pulse oximetry. Seven hammocks were used throughout the study, washed and disinfected every five days or whenever assigned to a new patient (Figure 2).

In the combined intervention group, neonates first underwent hydrotherapy for 15 minutes, followed immediately by hammock positioning for 120 minutes, following the same protocols described above. Consequently, the hydrotherapy group received 15 minutes of therapy daily, the hammock group 120 minutes daily, and the combined group 135 minutes daily.

#### Rationale for intervention duration and comparability

The duration of each intervention was determined based on physiological feasibility, safety evidence, and prior clinical practice. Hydrotherapy is limited to shorter exposure times (15 minutes) due to thermal regulation and water safety considerations, whereas hammock positioning allows prolonged sensory modulation and postural containment, justifying longer exposure. The combined intervention was designed to integrate both modalities sequentially without altering their established parameters. Comparisons among groups were based on within-group pre- and post-intervention pain changes rather than direct equivalence of exposure time, thereby allowing assessment of each modality’s analgesic effectiveness within its clinical context.

### Outcomes

The primary outcome was neonatal pain, assessed using the Neonatal Facial Coding System (NFCS) [16]. The NFCS was selected because it is one of the most widely validated and behaviorally specific instruments for assessing pain in preterm neonates, particularly in nonverbal populations and nonprocedural contexts. It has demonstrated strong construct validity, inter-rater reliability, and sensitivity to change in neonatal pain studies.

The NFCS measures pain through eight observable facial expressions. Each expression is scored as absent (0) or present (1), yielding a total score ranging from 0 (no pain indicators) to 8 (maximum pain expression). Scores ≥ 3 are considered indicative of clinically significant pain, a cutoff supported by validation studies and widely adopted in neonatal research. The facial movements assessed are: bulged forehead, squeezed eyes, deepened nasolabial furrow, parted lips, stretched mouth, pursed lips, taut tongue, and chin quiver [10].

Assessments were performed twice daily, five minutes before and immediately after each intervention, with neonates positioned in incubators or cribs pre-intervention and in the therapeutic position post-intervention. Each group underwent 225 pre- and 225 post-intervention evaluations, totaling 1,350 assessments across the study.

### Training of assessors

Pain assessments were conducted by four experienced physical therapists, all of whom underwent three days of standardized training to ensure consistency in intervention delivery and data collection. Inter-rater agreement was verified during training sessions using sample video recordings, ensuring uniform interpretation of NFCS criteria. In total, the study involved 225 interventions per group, with paired pain assessments before and after each session, resulting in 675 interventions and 1,350 NFCS analyses.

### Statistical analysis

Continuous variables, including NFCS scores, were analyzed using Student’s t-test. Categorical variables, defined as the presence or absence of pain (NFCS ≥ 3), were analyzed using Fisher’s exact test. A significance level of 5% (p < 0.05) was adopted.

## Results

The present study demonstrated that hydrotherapy, hammock positioning, and the combination of these two techniques were effective in reducing pain scores in preterm neonates.

A total of 45 preterm neonates were included in the analysis, with 15 allocated to each intervention group. The groups were comparable at baseline with respect to key demographic and clinical characteristics (Table 1). There were no statistically significant differences among the three groups regarding sex distribution (p = 0.84), mean gestational age (p = 0.78), birth weight (p = 0.91), postnatal age at enrollment (p = 0.83), or type of feeding (p = 0.89). The overall sample consisted of 24 male neonates (53.3%) and 21 female neonates (46.7%), with a mean gestational age of 32.4 ± 2.1 weeks and a mean birth weight of 1,720 ± 310 g. All neonates were receiving enteral feeding via an orogastric tube at the time of enrollment. These findings indicate that the intervention groups were clinically homogeneous at baseline.

**Table 1:** Demographic and clinical characteristics of preterm neonates by intervention group.

Variable	Hydrotherapy (n = 15)	Hammock Positioning (n = 15)	Combined Intervention (n = 15)	p-value
Sex, n (%)				0.84^1^
Male	8 (53.3)	7 (46.7)	9 (60.0)	
Female	7 (46.7)	8 (53.3)	6 (40.0)	
Gestational age (weeks), mean ± SD	32.6 ± 2.0	32.1 ± 2.3	32.5 ± 2.1	0.78^2^
Birth weight (g), mean ± SD	1,740 ± 295	1,695 ± 320	1,725 ± 315	0.91^2^
Postnatal age at enrollment (days), mean ± SD	9.4 ± 2.1	9.1 ± 2.4	9.6 ± 2.0	0.83^2^
Type of feeding, n (%)				0.89^1^
Exclusive breast milk	6 (40.0)	5 (33.3)	6 (40.0)	
Mixed feeding	9 (60.0)	10 (66.7)	9 (60.0)	
Use of orogastric tube, n (%)	15 (100)	15 (100)	15 (100)	—

Notes: ^1^ Fisher’s exact test.

2 One-way ANOVA.

Following hydrotherapy, neonates showed a significant reduction in pain, as evidenced by a decrease in NFCS scores from 3.98 ± 1.65 to 2.02 ± 1.06 (p < 0.0001; Table 2). Before hydrotherapy, 78.7% of neonates presented with pain, while after the intervention, only 28.4% had an NFCS score ≥ 3 (p < 0.0001; Table 3).

**Table 2:** Pain assessment measured using the NFCS scale in preterm infants before and after interventions.

Procedures	Before the intervention (n = 225) (95% CI)	After the intervention (n = 225) (95% CI)	p
Hydrotherapy	3.98 ± 1.65 (2.33 – 5.63)	2.02 ± 1.06 (0.96 – 3.08)	<0.0001
Hammock positioning therapy	3.01 ± 1.49 (1.52 – 4.50)	2.03 ± 1.11 (0.92 – 3.14)	<0.0001
Hydrotherapy + Hammock	3.40 ± 1.59 (1.81 – 4.99)	1.87 ± 0.87 (1.00 – 2.74)	<0.0001

**Notes:** NFCS, Neonatal Facial Coding System; Values expressed as mean ± standard deviation (M ± SD); CI, confidence interval; n, number of pain measurements; p values obtained using Student’s *t*-test.

**Table 3:** Evaluation of the number of positive pain assessments in preterm infants using the NFCS scale, before and after procedures.

Procedure	Before Intervention (n)	After Intervention (n)	p
Hydrotherapy	177 (78.7%)	64 (28.4%)	0.0001
Hammock Positioning	126 (56.0%)	73 (32.4%)	0.0001
Hydrotherapy + Hammock Positioning	150 (66.7%)	42 (18.7%)	0.0001

**Notes:** NFCS, Neonatal Facial Coding System; n, number of pain measurements; Values expressed as absolute numbers and percentages of patients with pain; *p* values calculated using Fisher’s exact test.

For neonates undergoing hammock positioning, mean pain scores decreased from 3.01 ± 1.49 to 2.03 ± 1.11 after the intervention (p < 0.0001; Table 2). Prior to this intervention, 56% of neonates exhibited pain, whereas after the intervention, only 32.4% did (p < 0.0001; Table 3).

When neonates received hydrotherapy followed by hammock positioning, pain scores also improved, decreasing from 3.40 ± 1.59 to 1.87 ± 0.87 (p < 0.0001; Table 2). In this group, 66.7% of neonates initially presented with pain, compared with only 18.7% after the intervention (p < 0.0001; Table 3).

Exploratory analyses did not demonstrate significant associations between demographic variables (sex, gestational age, birth weight, postnatal age, or type of feeding) and the magnitude of pain reduction within any intervention group (all p > 0.05), suggesting that the analgesic effects observed were independent of baseline neonatal characteristics.

Consistent with the study objective, statistical analyses were limited to within-group comparisons before and after each intervention. Direct between-group comparisons of intervention efficacy were not performed.

## Discussion

The most important finding of this randomized clinical trial is that hydrotherapy, hammock positioning, and the combination of the two interventions significantly reduced pain in preterm neonates, as measured by the Neonatal Facial Coding System (NFCS). Importantly, pain scores decreased to values below the validated cut-off for pain, indicating not only statistical but also clinical relevance. Across 1,350 pain assessments performed in 45 preterm neonates over 15 consecutive days, these findings provide robust evidence supporting the effectiveness of these non-pharmacological interventions in neonatal pain management.

Both interventions were effective when applied individually, and the combined intervention produced comparable analgesic benefits, suggesting that hydrotherapy and hammock positioning act as complementary comfort strategies rather than competing modalities. The consistent reduction in NFCS scores before and after each intervention supports their role as reliable adjuncts to routine neonatal care during painful or stressful aspects of routine neonatal care.

The present results are consistent with prior research demonstrating the benefits of hammock positioning. Queiroz et al. (2018) [17], for instance, showed that both hammock and prone positioning reduced NFCS scores in 20 preterm infants, although their study included only 40 pain assessments. Similarly, Giamellaro et al. (2018) [18] observed a significant reduction in pain intensity among ventilated neonates using hammock positioning, but their findings were based on only 16 measurements. Ribas et al. (2019) [19] reported improvement using the Premature Infant Pain Profile (PIPP), but not the NFCS, highlighting how outcomes may vary depending on the pain assessment instrument employed. Conversely, Jesus et al. (2018) [14] did not detect significant benefits, which may be attributable to methodological differences, shorter intervention duration, and a smaller sample size.

With respect to hydrotherapy, these findings align with those of Vignochi et al. (2010) [20], Silva et al. (2016) [21], and Cecconello et al. (2010) [22], all of whom demonstrated significant reductions in neonatal pain or stress indicators following aquatic interventions. Compared with these studies, the present trial included a larger number of repeated pain measurements, strengthening the reliability of the observed effects. In contrast, de Oliveira Tobinaga et al. (2016) [23] reported improvements in physiological stress markers, such as reduced salivary cortisol and stabilized vital signs, without significant changes in NIPS pain scores, underscoring the complexity of neonatal pain assessment and the importance of using validated, behavior-based tools such as the NFCS.

Hydrotherapy and hammock positioning offer several practical advantages: they are low-cost, easy to implement, and associated with minimal risk when performed by trained professionals. Given the well-documented risks of pharmacological analgesia in neonates — including respiratory depression, feeding intolerance, urinary retention, and potential long-term neurodevelopmental effects [24,25], non-pharmacological strategies should be prioritized whenever possible. Morphine, although frequently used in NICUs, has been associated with later behavioral disturbances, including anxiety, depression, and attention deficits [26]. Even non-opioid analgesics are prescribed cautiously in this population, with acetaminophen remaining the only widely accepted option [27].

Established non-pharmacological interventions, such as skin-to-skin contact, breastfeeding, oral sucrose, and non-nutritive sucking, have demonstrated efficacy in reducing procedural pain in neonates [[28], [29], [30]]. The present study further expands this evidence base by supporting hydrotherapy and hammock positioning as additional, evidence-based options for neonatal comfort and pain modulation.

The strengths of this study include the large number of pain assessments (n = 1,350), the use of a validated and widely recognized pain assessment tool (NFCS), and the evaluation of both individual and combined non-pharmacological interventions. However, several limitations should be acknowledged. First, the study was conducted at a single center, which may limit generalizability. Second, although standardized training was provided, facial expression–based assessments are inherently subject to some degree of inter-rater variability. Third, the analysis focused on short-term pain outcomes, and long-term effects on neurodevelopment, stress regulation, and behavioral outcomes were not evaluated. Future studies should therefore investigate whether repeated exposure to these interventions yields sustained benefits beyond the neonatal period.

Overall, the findings of this study support the incorporation of hydrotherapy and hammock positioning into standardized pain management protocols in neonatal intensive care units. Future research should explore the potential synergistic effects of combining these interventions with other evidence-based non-pharmacological strategies, such as kangaroo care or oral sucrose, and assess long-term outcomes, including neurodevelopmental trajectories and caregiver – infant bonding.

In conclusion, hydrotherapy and hammock positioning, applied individually or in combination, are effective, safe, and feasible strategies for reducing pain in preterm neonates. Their integration into routine NICU practice may reduce reliance on pharmacological analgesia and contribute to safer, more holistic neonatal care.

## Funding

None.

## Authors’ contributions

Ana Talita Vasconcelos Arcanjo Ribeiro da Silva: Conceptualization, data curation, formal analysis, investigation, methodology, validation, visualization, writing original draft, writing review & editing.

Francisco Placido Nogueira Arcanjo: Project Administration, conceptualization, data curation, formal analysis, investigation, methodology, validation, visualization, writing original draft, writing review & editing.

Jeferson de Sousa Justino: Data curation, formal analysis, investigation, methodology, validation, visualization, writing original draft, writing review & editing.

Lizandro de Andrade Teles: Data curation, formal analysis, investigation, methodology, validation, visualization, writing original draft, writing review & editing.

Ana Kelly Melo de Aquino: Data curation, formal analysis, investigation, methodology, validation, visualization, writing original draft, writing review & editing.

All authors participated in validation, visualization, writing the original draft, writing review & editing.

## Data availability statement

The contents underlying the research text are included in the manuscript.

## Conflicts of interest

The authors declare no conflicts of interest.

## References

[1] World Health Organization (WHO) (2023). https://www.who.int/en/news-room/fact-sheets/detail/preterm-birth.

[2] Perry M., Tan Z., Chen J., Weidig T., Xu W., Cong XS. (2018). Neonatal pain: perceptions and current practice. Crit Care Nurs Clin North Am.

[3] Fitzgerald M. (2005). The development of nociceptive circuits. Nat Rev Neurosci.

[4] Hartley C., Slater R. (2014). Neurophysiological measures of nociceptive brain activity in the newborn infant–the next steps. Acta Paediatr.

[5] Sociedade Brasileira de Pediatria (SBP) (2025). https://www.sbp.com.br/imprensa/detalhe/news/abordagem-da-dor-no-recem-nascido/.

[6] Raja S.N., Carr D.B., Cohen M., Finnerup N.B., Flor H., Gibson S. (2020). The revised international association for the study of pain definition of pain: concepts, challenges, and compromises. Pain.

[7] van Dokkum N.H., de Kroon M.L., Reijneveld S.A., Bos AF. (2021). Neonatal stress, health, and development in preterms: a systematic review. Pediatrics.

[8] Zhao T., Griffith T., Zhang Y., Li H., Hussain N., Lester B. (2022). Early-life factors associated with neurobehavioral outcomes in preterm infants during NICU hospitalization. Pediatr Res.

[9] McLean M.A., Nakajima L., Chau C.M., Weinberg J., Synnes A.R., Miller S.P. (2023). Cortisol levels are related to neonatal pain exposure in children born very preterm at age 18 months in two independent cohorts. Paediatr Neonatal Pain.

[10] Grunau R.V., Craig KD. (1987). Pain expression in neonates: facial action and cry. Pain.

[11] Lawrence J., Alcock D., McGrath P., Kay J., MacMurray S.B., Dulberg C. (1993). The development of a tool to assess neonatal pain. Neonatal Netw.

[12] Dos Anjos F.R., Nakato A.M., Hembecker P.K., Nohama P., Sarquis A.L. (2022). Effects of hydrotherapy and tactile-kinesthetic stimulation on weight gain of preterm infants admitted in the neonatal intensive care unit. J Pediatr (Rio J).

[13] Novakoski K.R., Valderramas S.R., Israel V.L., Yamaguchi B., Andreazza MG. (2018). Back to the liquid environment: effects of aquatic physiotherapy intervention performed on preterm infants. Rev Bras Cineantropom Desempenho Hum.

[14] Jesus V.R., Oliveira P.M., Azevedo VM. (2018). Effects of hammock positioning in behavioral status, vital signs, and pain in preterms: a case series study. Braz J Phys Ther.

[15] Sousa A.G., Carvalho E.E., Souza J.M., Mendonça J.F., Moran C.A., Gomes ÉL. (2021). Effects of the hammock method on preterm newborns: systematic review and meta-analysis. Childs Health.

[16] Grunau R.E., Oberlander T., Holsti L., Whitfield MF. (1998). Bedside application of the neonatal facial coding system in pain assessment of premature neonates. Pain.

[17] Queiroz C.M., Santos-de-Araújo A.D., Silva L.M., Silva-Júnior J.A., Bassi-Dibai D., Santos-de-Souza C.T. (2018). Repercussions on the neonate of using hammock positioning and prone positioning. Rev Bras Ter Intensiva.

[18] Giamellaro A., Oliveira E.A., Rodrigues E.C., Andrade NV. (2018). Avaliação das variáveis cardiorrespiratórias após o uso da terapia de rede de descanso em recém-nascidos pré-termo ventilados mecanicamente e sob oxigenoterapia. Arq Med Hosp Fac Ciênc Med St Casa São Paulo.

[19] Ribas C.G., Andreazza M.G., Neves V.C., Valderramas S. (2019). Effectiveness of hammock positioning in reducing pain and improving sleep-wakefulness state in preterm infants. Respir Care.

[20] Vignochi C.M., Teixeira P.P., Nader SS. (2010). Effect of aquatic physical therapy on pain and state of sleep and wakefulness among stable preterm newborns in neonatal intensive care units. Rev Bras Fisioter.

[21] Silva H.A., Silva K.C., Reco M.O., Costa A.S., Soares-Marangoni D.A., Merey LS. (2016). Efeitos fisiológicos da hidroterapia em balde em recém-nascidos prematuros [Physiological effects of bucket hydrotherapy for premature newborns]. Rev Bras Ter Intensiva.

[22] Cecconello B.W., Borba E.O., Lisboa D.D., Cecconello WW. (2010). Efeitos da hidroterapia na dor e nos sinais vitais de recém-nascidos prematuros internados em uma UTI neonatal [Effect of hydrotherapy in pain and vital signs of preterm newborns in a neonatal ICU]. Rev Bras Ter Intensiva.

[23] de Oliveira Tobinaga W.C., de Lima Marinho C., Abelenda V.L., de Sá P.M., Lopes AJ. (2016). Short-term effects of hydrokinesiotherapy in hospitalized preterm newborns. Rehabil Res Pr.

[24] Puia-Dumitrescu M., Comstock B.A., Li S., Heagerty P.J., Perez K.M., Law J.B. (2021). Assessment of 2-year neurodevelopmental outcomes in extremely preterm infants receiving opioids and benzodiazepines. JAMA Netw Open.

[25] McPherson C., Miller S.P., El-Dib M., Massaro A.N., Inder TE. (2020). The influence of pain, agitation, and their management on the immature brain. Pediatr Res.

[26] Anand KJ. (2021). Neonatal opioids and preschool outcomes. Pediatr Res.

[27] Ohlsson A., Shah PS. (2020). Paracetamol (acetaminophen) for prevention or treatment of pain in newborns. Cochrane Database Syst Rev.

[28] Stevens B., Yamada J., Lee G.Y., Ohlsson A. (2013). Sucrose for analgesia in newborn infants undergoing painful procedures. Cochrane Database Syst Rev.

[29] Koukou Z., Theodoridou A., Taousani E., Antonakou A., Panteris E., Papadopoulou S.S. (2022). Effectiveness of non-pharmacological methods, such as breastfeeding, to mitigate pain in NICU Infants. Child (Basel).

[30] Queirós I., Moreira T., Pissarra R., Soares H., Guimarães H. (2023). Non-pharmacological management of neonatal pain: a systematic review. Minerva Pediatr (Torino).

